# Household-Based HIV Counseling and Testing as a Platform for Referral to HIV Care and Medical Male Circumcision in Uganda: A Pilot Evaluation

**DOI:** 10.1371/journal.pone.0051620

**Published:** 2012-12-13

**Authors:** Henry Tumwebaze, Elioda Tumwesigye, Jared M. Baeten, Ann E. Kurth, Jennifer Revall, Pamela M. Murnane, Larry W. Chang, Connie Celum

**Affiliations:** 1 Integrated Community Based Initiatives, Kabwohe, Uganda; 2 Department of Global Health, University of Washington, Seattle, Washington, United States of America; 3 Department of Medicine, University of Washington, Seattle, Washington, United States of America; 4 Department of Epidemiology, University of Washington, Seattle, Washington, United States of America; 5 College of Nursing, New York University, New York, New York, United States of America; 6 Department of Medicine, Johns Hopkins University, Baltimore, Maryland, United States of America; 7 Department of International Health, Johns Hopkins University, Baltimore, Maryland, United States of America; Asociacion Civil Impacta Salud y Educacion, Peru

## Abstract

**Background:**

Combination HIV prevention initiatives incorporate evidence-based, biomedical and behavioral interventions appropriate and acceptable to specific populations, aiming to significantly reduce population-level HIV incidence. Knowledge of HIV serostatus is key to linkages to HIV care and prevention. Household-based HIV counseling and testing (HBCT) can achieve high HIV testing rates. We evaluated HBCT as a platform for delivery of combination HIV prevention services in sub-Saharan Africa.

**Methods:**

We conducted HBCT in a semi-urban area in southwestern Uganda. All adults received standard HIV prevention messaging. Real-time electronic data collection included a brief risk assessment and prevention triage algorithm for referrals of HIV seropositive persons to HIV care and uncircumcised HIV seronegative men with multiple sex partners to male circumcision. Monthly follow-up visits for 3 months were conducted to promote uptake of HIV care and male circumcision.

**Results:**

855 households received HBCT; 1587 of 1941 (81.8%) adults were present at the HBCT visit, 1557 (98.1% of those present) were tested and received HIV results, of whom, 46.5% were men. A total of 152 (9.8%) were HIV seropositive, for whom the median CD4 count was 456 cells/µL, and 50.7% were newly-identified as HIV seropositive. Three months after HBCT, 88.5% of HIV seropositive persons had attended an HIV care clinic; among those with CD4 counts <250 cells/µL, 71.4% initiated antiretroviral therapy. Among 123 HIV seronegative men with an HIV+ partner or multiple partners, 62.0% were circumcised by month 3.

**Conclusions:**

HBCT achieves high levels of knowledge of HIV serostatus and is an effective platform for identifying at-risk persons and achieving higher uptake of HIV prevention and care services through referrals and targeted follow-up than has been accomplished through other single focus strategies.

## Introduction

More than two-thirds of the estimated 33.4 million persons living with HIV reside in sub-Saharan Africa, the region hardest hit by the pandemic [1]. No single intervention offers a “magic bullet” for HIV prevention; instead, a multi-component, integrated package of evidence-based, effective, biomedical and behavioral interventions must be tailored to be appropriate, acceptable, and deliverable to subpopulations at highest risk for HIV transmission.

In the past several years, randomized clinical trials have demonstrated that medical male circumcision (MC) of HIV seronegative men [2], [3], [4] and antiretroviral therapy (ART) initiation by HIV seropositive persons [5], [6] are highly effective HIV prevention strategies. To move from the high levels of efficacy observed in these clinical trials to population-level effectiveness requires new approaches to deliver HIV prevention and care services with high coverage of those at greatest risk for HIV transmission or acquisition.

Large scale implementation of evidenced-based HIV care and prevention interventions requires near-universal HIV testing to identify HIV seropositive persons early in order to initiate ART promptly when eligible and HIV negative persons for linkages to medical male circumcision and other effective interventions. However, only a minority of African adults know their serostatus [7], in part due to limited access to and utilization of facility-based HIV testing. Recent evaluations of household-based voluntary counseling and testing (HBCT) have demonstrated that district-wide testing of hundreds of thousands of persons is highly acceptable, logistically feasible, and cost-effective relative to other strategies for HIV testing, and may possibly help reduce stigma and risk behaviors [8], [9], [10]. A systematic review and meta-analysis of home-based testing in Africa found a pooled proportion of 83% of persons tested across 16 studies conducted in 5 countries (Uganda, Malawi, Kenya, South Africa and Zambia) [11]. HBCT is potentially an effective method to identify key at-risk populations, including HIV seropositive persons who are unaware of their status [12], [13] who would receive clinical and prevention benefits of ART and high-risk HIV uninfected individuals who would benefit from evidence-based HIV prevention services such as MC.

The UNAIDS goal of achieving universal access to HIV prevention and care requires universal access to HIV testing. HBCT could serve as a platform to provide near-universal voluntary testing and knowledge of HIV status and would have greatest impact if HBCT was effective in achieving linkages to HIV care and evidence-based HIV prevention interventions. We built on the success of previous HBCT projects with a pilot study that adapted HBCT by incorporating mobile phone data collection with an electronic triage algorithm to facilitate linkage to HIV prevention and care, targeted and tailored to key subpopulations.

## Methods

### Study Setting

The study was conducted between November 2010 and July 2011 in Kabwohe parish, a mixed rural and semi-urban area in southwestern Uganda. The area includes a single government health unit and a private clinical center. HBCT was previously conducted in this area between 2004 and 2007 [8].

### Research Design and Participants

The overall strategy focused on HBCT as a platform for provision of HIV serostatus, individualized assessment of HIV acquisition or transmission risk, and referral of HIV seropositive persons to HIV care and targeted referral of HIV seronegative men with multiple partners to MC services. All adults aged ≥18 years residing in the study area who were able to provide informed consent were eligible for the study. Before study initiation, the study team conducted local stakeholder consultations and introduced the purpose and procedures of the study and the members of the study team.

### Ethics Statement

The study protocol was approved by the AIDS Research Committee of the Uganda National Council for Science and Technology, the New York University School of Medicine Institutional Review Board, and the Human Subjects Review Committee at the University of Washington. Participants provided written informed consent.

### Study Procedures

Community outreach workers (an existing health cadre in Uganda) approached all households to notify that HBCT would be offered in the area, as previously described [8]. Trained and experienced HBCT study staff then conducted HBCT, beginning with providing HIV education on a house-to-house basis. After obtaining assent of the head of household, the purpose of the study was explained to the household and standardized HIV prevention messaging was provided. Household information, including location and age and gender of adults living in the household, was recorded. A confidential workspace was prepared in or behind the home. Confidential written informed consent was then obtained from each participating adult. Individuals completed a structured, face-to-face questionnaire covering demographic characteristics, sexual behaviour, and prior HIV testing. A mobile electronic data collection system (using an open-source Android software platform on Google phones) was employed for data capture (eMOCHA, Baltimore, MD) [14]. Individual HIV counselling and testing was then conducted, following the Uganda national serial rapid testing algorithm using blood obtained by fingerstick: Determine (Abbott Laboratories) followed by Statpak (Chembio Diagnostics) for confirmation of positive results and Unigold (Trinity Biotech) as a tie breaker, if needed. The study site enrolled in an external quality assurance (EQA) program for HIV rapid testing and performed routine internal quality control. Quality assurance testing with a prior HBCT project demonstrated >99% agreement between rapid test results, using this same testing algorithm, and blood test results using ELISA [8]. Post-test counselling was performed, and a card with HIV status was offered to facilitate establishment of care at local HIV clinics. Couples were offered facilitated mutual disclosure of HIV test results.

### Referral for HIV Care and Prevention Services with Targeted Follow-up

For the purposes of the study, two subpopulations were identified for referral and targeted follow-up for HIV care and prevention services: 1) HIV seropositive individuals were referred to local HIV clinics for ART eligibility assessment, and 2) uncircumcised HIV seronegative men age >18 were referred for voluntary medical MC. However, follow-up visits at 1, 2, and 3 months were targeted to HIV- men meeting specific behavioral criteria, specifically reporting >1 sexual partner (>2 if polygamous) or a known HIV seropositive partner, to determine the proportion of uncircumcised HIV seronegative men with higher behavioral risk who were circumcised after referral. HIV prevention and care services were available in the study communities through public and community-based organizations. The mobile electronic data collection device automatically triggered research staff in real-time to recognize that additional questionnaires, counseling modules and follow-up were needed for individuals from these specific subpopulations based on pre-programmed algorithms following administration of a core set of questions to all subjects.

For HIV seropositive persons, a questionnaire was administered by HBCT staff, covering previous uptake of HIV care services. A blood sample was obtained for CD4 measurement using flow cytometry (BD FACSCalibur, BD Biosciences); results were returned to the participant at home approximately 1 week later. Individualized, targeted counseling about HIV and the importance of accessing HIV care was conducted. Information on local resources for persons with HIV and a written referral to local HIV care clinics was provided. Follow-up visits were scheduled at 1, 2, and 3 months, at which time a standardized questionnaire about accessing HIV care services and targeted counselling about local clinics providing HIV care were conducted; after the first month, follow-up visits were discontinued for those taking antiretroviral therapy (ART). At the time of the study, Uganda national guidelines recommended ART initiation for those with CD4 counts <250 cells/µL.

For uncircumcised higher-risk HIV seronegative men, a standardized questionnaire was administered and counselling about MC for HIV prevention was done by HBCT staff. A written referral to a local clinic at which MC could be obtained without charge was provided. Targeted follow-up visits for HIV- men with a known HIV seropositive partner or multiple partners were prompted by the mobile device and scheduled at 1, 2, and 3 months, at which time a staff member administered a standardized questionnaire about MC and provided referral information again, if necessary.

All participants completed a face-to-face questionnaire about their experience of being in the study, including questions about potential social harm, which was asked at each follow-up visit of HIV-infected persons, breaches in confidentiality, and participant experience with the provision of referrals within the context of HBCT and the use of the electronic data collection device. At the beginning, during, and at the end of the study, the study team organised meetings to share experiences and challenges with the pilot.

### Data Analysis

Uptake of HBCT was ascertained with the number of households and adults enumerated, contacted during HBCT, and the number and proportion of adults who accepted testing, the proportion HIV seropositive, and the proportion of newly-identified infections. Demographic characteristics of participants were described, including medians and interquartile ranges for continuous variables and proportions for categorical variables. Among those referred for HIV care, ART initiation, and male circumcision, the cumulative probability of referral uptake was estimated with the Kaplan-Meier method. Analyses were performed using SAS version 9.2 (SAS Institute, Cary, NC).

## Results

### Participant Characteristics

A total of 855 households were visited (Table 1). Of 1941 adults residing in these households, 1587 (81.8%) were present and 1557 (98.1% of those present) underwent HIV testing and received their results. Nearly half (46.5%) of those tested were men and 29% had not been tested previously. Participant characteristics are detailed in Table 2. Among the study population, the median number of sexual partners was one, about half were married in monogamous relationships, and <20% used a condom with last sex. Overall, 84.7% of 672 HIV seronegative men were uncircumcised.

**Table 1:** Home-based HIV counseling and testing in southwestern Uganda.

**Households approached for HBCT**	855
**Adults living in the households**	1941
**Adults present (% of those living in the households)**	1587 (81.8%)
**Total adults tested (% of those present)**	1558 (98.2%)
**Received results (% of those tested)**	1557 (99.9%)
Female	834 (53.6%)
Male	724 (46.5%)
First time tested	453 (29.1%)
**HIV seropositive (% of tested & received results)**	152 (9.8%)
Newly-identified infections (% of positives)	77 (50.7%)
**Couples**	
Adults reporting a partner in the home (% of tested)	544 (34.9%)
Partner tested (% of those reporting partner in home)	328 (60.3%)
**Couples tested**	164
Both partners disclosed to each other	161 (98.2%)
HIV serodiscordant (% of couples)	15 (9.1%)
HIV concordant positive (% of couples)	15 (9.1%)
HIV concordant negative (% of couples)	134 (81.7%)

HBCT = home-based HIV counseling and testing.

**Table 2:** Population characteristics.

		FEMALES		MALES		
		HIV Seropositive N = 100	HIV seronegative N = 734	HIV seropositive N = 52		HIV seronegative N = 672
**Age, years, median (IQR)**		28 (23–36)	25 (20–35)	30 (28–40)		25 (21–35)
**Years of education, median (IQR)**		6 (3–7)	7 (5–11)	7 (4–11)		9 (7–12)
**Marital Status**	Married monogamous*	53 (53.0%)	422 (57.5%)	37 (71.1%)		355 (52.8%)
	Married polygamous	0	0	4 (7.7%)		8 (1.2%)
	Divorced	20 (20.0%)	35 (4.8%)	1 (1.9%)		18 (2.7%)
	Widowed	17 (17.0%)	51 (6.9%)	2 (3.8%)		3 (0.4%)
	Single	10 (10.0%)	226 (30.8%)	8 (15.4%)		288 (42.9%)
**Current sex partners, median (IQR)**		1 (0–1)	1 (0–1)	1 (1–1)		1 (1–1)
**Condom use with last sex**	No	79 (79.0%)	543 (74.6%)	41 (78.8%)		467 (70.9%)
	Yes	17 (17.0%)	91 (12.5%)	11 (21.2%)		120 (18.2%)
	Don’t know	1 (1.0%)	10 (1.4%)	0		6 (0.9%)
	Decline to answer	3 (3.0%)	84 (11.5%)	0		66 (10.0%)
**Ever tested for HIV**	No	24 (24.0%)	155 (21.2%)	21 (40.4%)	253 (37.6%)	
	Yes	76 (76.0%)	575 (78.7%)	31 (59.6%)	414 (61.6%)	
	Decline to answer	0	1 (0.1%)	0	5 (0.7%)	
**Prior HIV testing result for persons** **who tested HIV+ through** **HBCT visit**	Negative	26 (34.2%)	572 (99.5%)	12 (38.7%)	411 (99.3%)	
	Positive	49 (64.5%)	0	19 (61.3%)	1 (0.2%)	
	Don’t know/decline to answer	1 (1.3%)	3 (0.5%)	0	2 (0.4%)	
**Among persons at HBCT visit** **who knew of their HIV** **infection**	Months knew status, median (IQR)	30.8 (8.8–53.5)	N/A	25 (8.5–54)	N/A	
	Knew HIV infected >1 year	38 (70.4%)	N/A	15 (71.4%)		N/A
	Had a CD4 count in past year	21 (55.3%)	N/A	10 (66.7%)		N/A
	Previously visited HIV clinic	48 (88.9%)	N/A	18 (85.7%)		N/A
**Uncircumcised men**		N/A	N/A	44 (84.6%)		570 (84.9%)

* Married monogamous represents civil as well as religious marriages.

Of the 1558 adults tested, 328 (21.1%) were in a partnership in which both members were tested through HBCT (Table 1); of these 164 couples tested, 15 were concordant HIV seropositive and 15 were serodiscordant (10 with the male partner seropositive and five with the female partner seropositive). Of the remaining adults tested, 551 reported that they have a partner but that s/he did not live in the home so was not tested through HBCT, 52 of the partners were not willing to be tested through HBCT, 142 reported that they had a partner who was not present and thus not tested through HBCT, 22 reported multiple partners, and 463 reported no partner.

### Characteristics of HIV Seropositive Persons and Couples

One hundred fifty-two individuals (9.8% of those tested) were HIV seropositive, of whom 100 (65.8%) were female (Table 2). The median age was 28 years for HIV seropositive women and 30 years for seropositive men. The median CD4 count of the 152 HIV seropositive individuals was 467 cells/µL (interquartile range [IQR] 330–687) overall (Table 3) and 479 cells/µL (IQR 330–715) among the 113 HIV seropositive persons who were not already on ART at the time of HBCT. Of the 152 HIV seropositive persons, 77 (50.7%) were newly-identified infections. Of the 75 who reported that they knew they were HIV seropositive at the HBCT visit, 54 (72.0%) were women; the median time reported of their knowledge of their HIV infection was 30 months (IQR 8.5–53.5 months). Sixty-six (88.0%) of those who were aware of their HIV infection prior to HBCT reported they had previously been to an HIV clinic. However, of the 28 HIV seropositive individuals with CD4<250 cells/µL and thus eligible for ART by Ugandan guidelines at the time of the study, only seven (25.0%) reported they were on ART and only half were on co-trimoxazole prophylaxis as recommended by Ugandan national guidelines.

**Table 3:** Follow-up.

		number (cumulative %* or IQR)
**HIV seropositive persons**	**Total N**	152
	**CD4 cells/µL, median (IQR)**	467 (330–687)
	**On ART by 3 month follow-up visit**	66 (43.9%)
	Initiated during follow up	34
	On at enrollment	32
	**On co-trimoxazole prophylaxis by 3 month follow-up visit**	131 (89.9%)
	Initiated during follow up	74
	On at enrollment	57
	**Visited HIV clinic by 3 month follow up visit**	133 (88.5%)
**HIV seropositive persons with CD4<250 cells/µL**	**Total N**	28
	**On ART by 3 month follow-up visit**	22 (78.6%)
	Initiated during follow up	15
	On at enrollment	7
	**On co-trimoxazole prophylaxis by 3 month follow-up visit**	24 (91.3%)
	Initiated during follow up	12
	On at enrollment	12
	**Visited HIV clinic by 3 month follow up visit**	25 (89.3%)
**Higher-risk HIV seronegative men referred for** **male circumcision**	**Total N**	123
	**Completed procedure by 3 month follow-up visit**	75 (62.0%)

* Cumulative % is estimated by the Kaplan-Meier method.

### Referral and Targeted Follow Up for HIV Care and Male Circumcision

**Figure 1:**
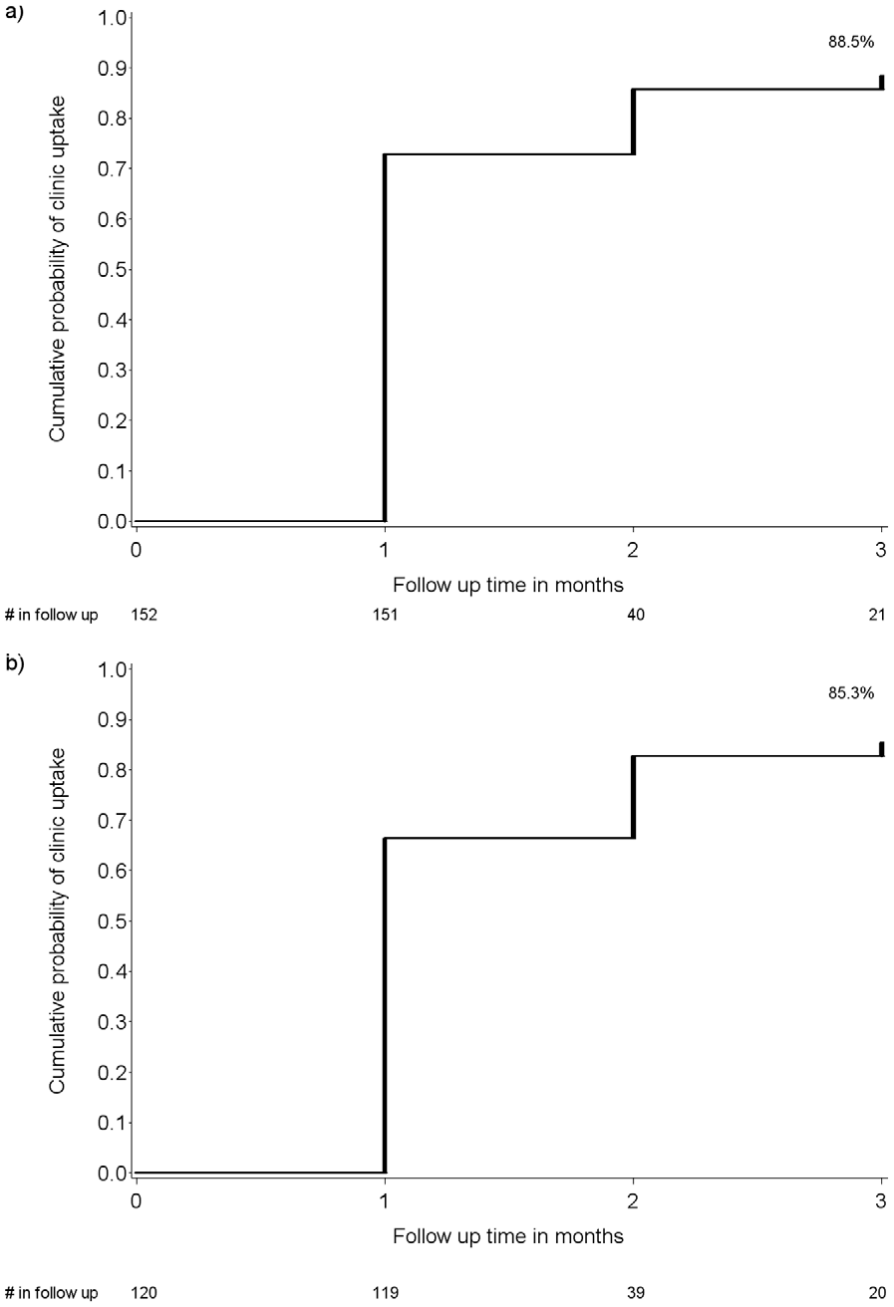
Cumulative probability of visiting an HIV clinic. Results are overall among a) all HIV seropositive persons (n = 152) and b) those not already on ART at baseline (n = 120). After month one, for those on ART, follow-up was discontinued. The cumulative probability of visiting an HIV clinic by 3 months was 88.5% overall and 85.3% among those not already on ART at baseline (Figure 1a and 1b, respectively).

**Figure 2:**
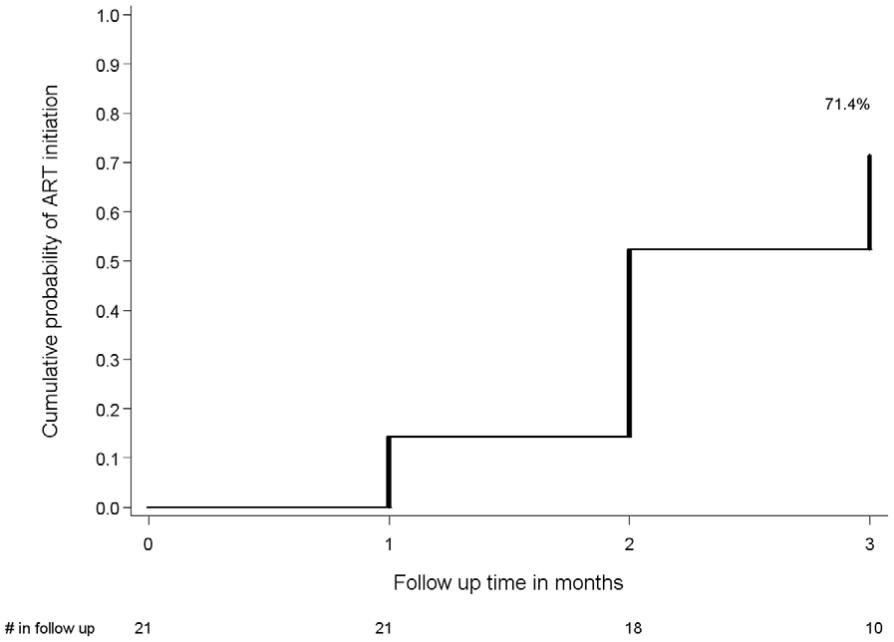
Cumulative probability of ART initiation, among HIV seropositive persons with a CD4 count <250 cells/µL and not on ART at baseline. Results are shown for the 21 HIV seropositive individuals who had a baseline CD4 count <250 cells/µL and were eligible for ART by Ugandan guidelines but not taking 7 ART at baseline. The cumulative probability of ART initiation by 3 months among those with CD4 count <250 cells/µL and not on ART at baseline was 71.4%.

Of the 152 HIV seropositive individuals identified, 3 (2.0%) were lost to follow-up during the three month follow-up period. The cumulative probability of visiting an HIV clinic by 3 months was 88.5% overall and 85.3% among those not already on ART at baseline (Figure 1a and 1b, respectively). The 19 who never visited an HIV clinic during the three months of follow-up primarily reported not attending due to being too busy (77.8%); 22.2% reported not wanting to be seen attending an HIV clinic. By three months, 66 (43.9%) were taking ART –32 who had been on ART at baseline plus an additional 34 who initiated during follow-up (Table 3). Among 28 individuals who had a baseline CD4 count <250 cells/µL and eligible for ART by Ugandan guidelines, seven were taking ART at baseline and 15 initiated during follow-up; the cumulative probability of ART initiation among those not on ART at baseline was 71.4% (Figure 2). Of the six HIV seropositive persons who met Ugandan ART eligibility and did not initiate ART during the study, three attended an HIV clinic.

**Figure 3:**
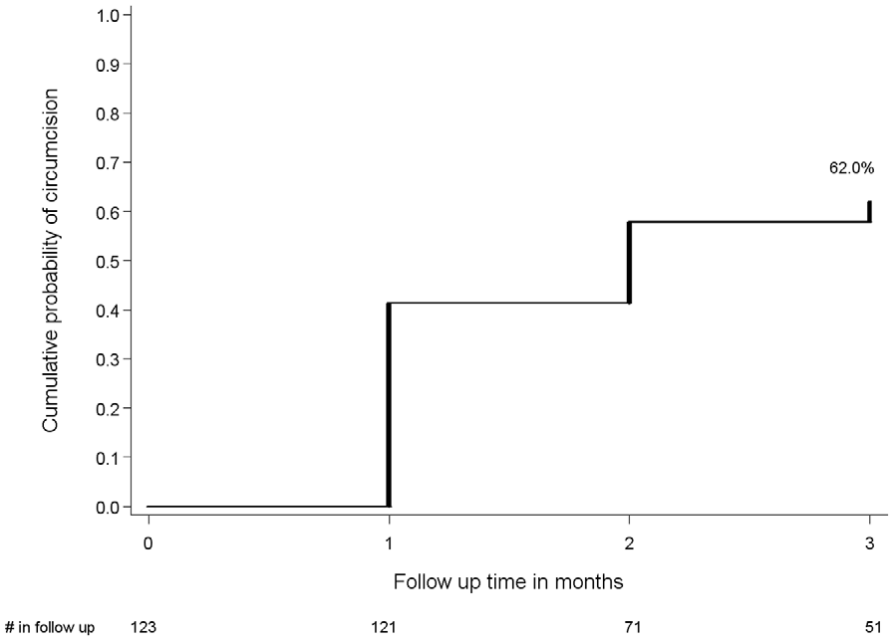
Cumulative probability of male circumcision, among HIV seronegative men with multiple partners or a known HIV seropositive partner. Results are shown for the 123 HIV seronegative men with >1 sexual partner (>2 if polygamous) or a known HIV seropositive partner who were referred for medical male circumcision. The cumulative probability of male circumcision by 3 months was 62.0%.

Of the 123 higher-risk uncircumcised HIV seronegative men referred for male circumcision, two (1.6%) were lost to follow-up, and 75 (62.0%) underwent circumcision during the three month follow-up period (Figure 3). Among the remaining 46 men who were referred but did not undergo circumcision, the median age was 27 years (IQR 22–32), only slightly older than men who were circumcised (median age 24, IQR 20–32) (p = 0.37). Fear of pain was the primary reported reason for not undergoing male circumcision (89.1%), with a minority reporting cultural norms (8.7%) and transportation costs (6.5%).

### Acceptability of HBCT and Electronic Data Collection

Overall, 99.3% of participants said they would recommend HBCT to others. Use of mobile phones for data collection was highly acceptable to participants: 96.0% reported it was “very” or “quite” acceptable. Fieldworkers found the mobile phone technology easy to use, that it reduced time in each home and was acceptable to persons approached for HBCT. Referrals to prevention and care services were positively received, with 96.9% of those referred finding it acceptable.

### Social Harms

Of the 1558 adults tested, six participants reported social harm, including five HIV-infected females who reported quarreling, verbal or physical abuse by their partners related to their HIV seropositive status. One of these episodes led to separation, two husbands prohibited their wives to go to the HIV clinic, and one continued in HIV care. In addition, one HIV seronegative male reported quarelling with his father who thought the antibiotics he took to facilitate wound healing after circumcision were antiretrovirals.

## Discussion

We piloted household HIV testing as a platform for combination HIV prevention in southwestern Uganda and demonstrated very high levels of HIV testing. Monthly follow-up visits over three months for HIV infected persons and for higher-risk uncircumcised HIV seronegative men facilitated very high uptake of HIV care and medical male circumcision referrals and addressed barriers to linkages. Half of HIV seropositive persons identified were unaware of their serostatus, and identified at an early stage (median CD4 count of 467 cells/µL) when they could benefit from counseling and engagement in HIV care.

Prior studies have shown that community-wide HBCT strategies can successfully test thousands of persons, with high acceptance, in multiple countries and in both rural and urban settings [8], [15], [16], [17], and with high accuracy of results using rapid HIV serologic tests [8], [17]. Our study found high uptake of HIV testing after community mobilization: 81.8% of those living in households were at home on the day of testing and 98.1% accepted testing received their results. The acceptability of mobile electronic data capture with HBCT was very high, and facilitated an integrated counseling and referral algorithm for referrals of HIV seropositive persons to HIV care and higher risk HIV seronegative men to male circumcision.

We found an HIV prevalence of 9.8%, comparable to the 9% HIV prevalence observed in southwestern Uganda from the 2011 Uganda AIDS Indicator Survey. A high proportion (50%) of those found to be HIV seropositive were unaware of their serostatus, however, only 25% were eligible for ART by Ugandan guidelines at the time of the HBCT visit and 11% of those who knew their HIV serostatus were not engaged in any HIV care and approximately 40% had not had a CD4 count in the prior year. A challenge of HIV testing programs, including HBCT programs, has been effective early engagement in HIV care. In a district-wide HBCT study from southwestern Uganda in 2004–7, only 11% initiated ART, most of whom had symptomatic HIV disease [8]. In recent HBCT projects in rural and urban Kenya, 42–54% of HIV-seropositive persons visited an HIV clinic within one month after HBCT [18]–[19]. HIV testing, even when done in a home-based setting, may not be sufficient to motivate early engagement in care, and follow-up visits may be needed to facilitate linkages to care. Challenges in linkage to HIV care occur at key transitions, such as from HIV testing to CD4 measurement or clinical staging to ART initiation, and need to be addressed to maximize the benefit of identifying HIV seropositive persons through HBCT [20], [21]. Earlier initiation of ART results in improved survival [22], and ART initiation is accompanied by a substantial decrease in HIV infectiousness [5], [6]. Recently, implementation of ART according to South African guidelines was associated with a reduction in population HIV incidence in rural KwaZulu-Natal [23], emphasizing the dual treatment and prevention potential of identification of HIV seropositive persons through testing with effective linkage to care and ART.

WHO guidelines recommend ART initiation at CD4 counts ≤350 cells/µL; however, in many African settings, the average CD4 count at the time of HIV diagnosis is significantly below 200 cells/µL [24], [25], indicating substantial missed opportunities for HIV testing, diagnosis, and linkage to care. HBCT identifies HIV seropositive persons with higher CD4 counts and asymptomatic disease [8], [16], [18], [19], median CD4 count was 467 cells/µL in our HBCT study. For HIV seropositive persons in our population who had CD4 counts <250 cells/µL, not taking ART at baseline, and eligible for ART under Ugandan guidelines at the time of the study, 71% initiated ART during the 3 months of study follow-up, and >85% of those who were HIV seropositive, regardless of CD4 count, were linked to HIV care. Thus, HBCT, by permitting identification of HIV seropositive persons earlier in the course of infection and coupled with lay counselor follow-up, offers a strategy for effective linkages to HIV care in order to realize the benefits of ART and other clinical and prevention interventions, including co-trimoxazole prophylaxis, isoniazid prophylaxis in areas with high tuberculosis prevalence, provision of bednets in malaria-endemic settings, risk reduction counseling, and condom promotion.

We provided referrals for HIV seronegative uncircumcised men in HBCT to medical MC services. Of the 123 men (22%) in HBCT who met our high risk criteria for targeted follow-up to assess uptake of MC, 62% reported having been circumcised by three months after HBCT (Figure 3) with 40% uptake in circumcision one month after HBCT. HBCT provides an opportunity to stimulate demand, facilitate referrals, and address men’s concerns about male circumcision. The high uptake of MC in this pilot is notable in a population where 85% of the HIV seronegative men are uncircumcised and given the challenges in achieving high levels of MC in subSaharan Africa including settings where MC trials were conducted [2], [26].

The public health benefits of HBCT also include behavior change from learning one’s HIV status and their partner’s serostatus. We demonstrated the feasibility of couples HIV testing and counseling (CHTC) in the context of HBCT. Notably, 21% were tested as couples, 99% mutually disclosed their results, and 10% of couples were HIV serodiscordant, who are a priority for HIV prevention interventions, [27], [28]. However, approximately one-third of the adults tested reported that their partner did not live in the household, for whom visits for HIV testing were not attempted. Additional CHTC strategies will be needed in parallel with HBCT in order to increase the proportion of couples who know and have disclosed their HIV serostatus to each other, as recommended by WHO [29].

The limitations of our study include that the study was conducted in a geographically small area and HBCT had been conducted in this area five years earlier. We did not independently verify self-reported outcomes of visiting an HIV clinic or MC provider. We did not have resources to randomize communities to HBCT versus standard HIV testing strategies to be able to directly compare linkage to HIV care and MC rates through HBCT with other testing strategies. The questionnaire did not fully capture HIV disclosure to partners who did not reside within the household. Follow-up in this pilot was limited to three months; future work should assess longer-term impact, particularly ongoing engagement in HIV care and treatment. Our study was limited to adults and therefore cannot address acceptability of HBCT and follow-up in those younger than 18 years of age, an important population at risk in sub-Saharan Africa.

In summary, this pilot evaluation from rural southwestern Uganda demonstrates that HBCT achieves high levels of knowledge of HIV serostatus and is an acceptable, effective platform for identifying at-risk persons and achieving high uptake of linkages to HIV care and MC services through targeted referrals with rare social harms reported. Future studies in larger populations from diverse settings are necessary to assess the generalizability and potential impact of this combination HIV prevention strategy.
